# Resonance Raman signature of intertube excitons in compositionally-defined carbon nanotube bundles

**DOI:** 10.1038/s41467-018-03057-7

**Published:** 2018-02-12

**Authors:** Jeffrey R. Simpson, Oleksiy Roslyak, Juan G. Duque, Erik H. Hároz, Jared J. Crochet, Hagen Telg, Andrei Piryatinski, Angela R. Hight Walker, Stephen K. Doorn

**Affiliations:** 1 Engineering Physics Division, National Institute of Standards and Technology (NIST), Gaithersburg, MD 20899 USA; 2 Department of Physics, Astronomy, and Geosciences, Towson University, Towson, MD 21252 USA; 3 Physics and Engineering Physics, Fordham University, Bronx, NY 10458 USA; 4 Chemistry Division, Physical Chemistry and Applied Spectroscopy, Los Alamos National Laboratory, Los Alamos, NM 87545 USA; 5 Center for Integrated Nanotechnologies, Los Alamos National Laboratory, Los Alamos, NM 87545 USA; 6 Theoretical Division, Los Alamos National Laboratory, Los Alamos, NM 87545 USA

## Abstract

Electronic interactions in low-dimensional nanomaterial heterostructures can lead to novel optical responses arising from exciton delocalization over the constituent materials. Similar phenomena have been suggested to arise between closely interacting semiconducting carbon nanotubes of identical structure. Such behavior in carbon nanotubes has potential to generate new exciton physics, impact exciton transport mechanisms in nanotube networks, and place nanotubes as one-dimensional models for such behaviors in systems of higher dimensionality. Here we use resonance Raman spectroscopy to probe intertube interactions in (6,5) chirality-enriched bundles. Raman excitation profiles for the radial breathing mode and G-mode display a previously unobserved sharp resonance feature. We show the feature is evidence for creation of intertube excitons and is identified as a Fano resonance arising from the interaction between intratube and intertube excitons. The universality of the model suggests that similar Raman excitation profile features may be observed for interlayer exciton resonances in 2D multilayered systems.

## Abstract (web summary)

Bundles of single-wall carbon nanotubes with enriched chirality can be used as model systems for exploring exciton physics in low-dimensional nanostructures. Here, the authors use resonant Raman spectroscopy to probe intertube interactions in bundles of (6,5)-enriched carbon nanotubes, and observe a Fano resonance arising from coupling between intertube and intratube excitons.

## Introduction

Photonics and optoelectronics of heterostructured low-dimensional nanomaterials systems are poised to rapidly advance as synthesis and assembly methods continue to progress. As a result, multiple material types and compositions can be paired in well-defined interaction geometries to give rise to emergent optical behaviors. Examples include van der Waals heterostructures of 2D materials including graphene, transition metal dichalcogenides, and hexagonal boron nitride^1–3^. Interlayer interactions in appropriately paired materials can give rise to such phenomena as interlayer excitons^4–6^ and exciton condensates^7^. Graphene bilayers interacting with defined twist angles also give rise to new optical resonances^8,9^ and heterostructures with 0-D semiconducting quantum dots can create plasmon-exciton interactions that alter photon emission statistics^10^.

Such defined compositions and interaction geometries can be extended to 1D systems as well. In particular, advances in single-wall carbon nanotube (SWCNT) separations are providing multiple routes to samples that are highly enriched in a single SWCNT structure or chirality [defined by the indices (*n*, *m*)]^11–13^. The ready availability of such chirally-enriched material provides a means to probe intertube interactions defined by specific chiralities. Crochet et al.^14^ provided evidence of intertube excitons arising from tunneling of correlated electron-hole pairs between closely interacting SWCNTs within small bundles enriched in the (6,5) chirality. This possibility expands the range of exciton physics in SWCNTs, while such compositionally-defined bundles are also of growing interest in solar energy harvesting^15,16^. In that context, emergence of intertube excitons has potential to impact exciton transport mechanisms in such networked assemblies^17,18^. Furthermore, intertube excitons in SWCNTs may serve as 1D anaologues of interlayer excitons in 2D systems. Characterization of these intertube excitons, however, has been limited to observation of red shifts in absorption spectra that are not adequately accounted for by electrodynamic effects, and energy splittings derived from poorly resolved photoluminescence (PL) spectra^14^. Beyond PL and absorption as spectroscopic probes, resonance Raman spectroscopy and excitation profiling of SWCNTs can provide a sensitive probe of their electronic structure^19–21^ and the effects of environmental interactions^22–24^. Resonance Raman excitation profiles (REPs) obtained on defined-composition SWCNT bundles can therefore be a powerful means to further study and establish the origins and characteristics of such intertube excitons.

Here we present a resonance Raman study of emergent exciton behavior arising in compositionally-enriched bundles of SWCNTs. Previous REP studies of SWCNT bundles have been limited to compositions consisting of mixed SWCNT chiralities^20,22,23^. While such earlier work confirmed expectations of red-shifting and broadening of the associated excitonic transitions in bundled systems^25–27^, no behaviors emerging from specific intertube interactions were found. In this work, we obtain REPs for a series of size-controlled SWCNT bundles that have been highly enriched in the (6,5) chirality. We find that specific (6,5)–(6,5) intertube interactions in such compositionally-defined bundles lead to the emergence of a previously unobserved sharp resonance feature that is superimposed on the intrinsic SWCNT REP. We show this sharp resonance (linewidth <10 meV) can be understood as arising from an interaction between a bright intratube exciton and a dark intertube exciton. We present a model for this interaction that provides a quantitative fit to the experimental data, yielding additional characteristics of the intertube exciton.

## Results

### Raman spectroscopy

**Fig. 1:**
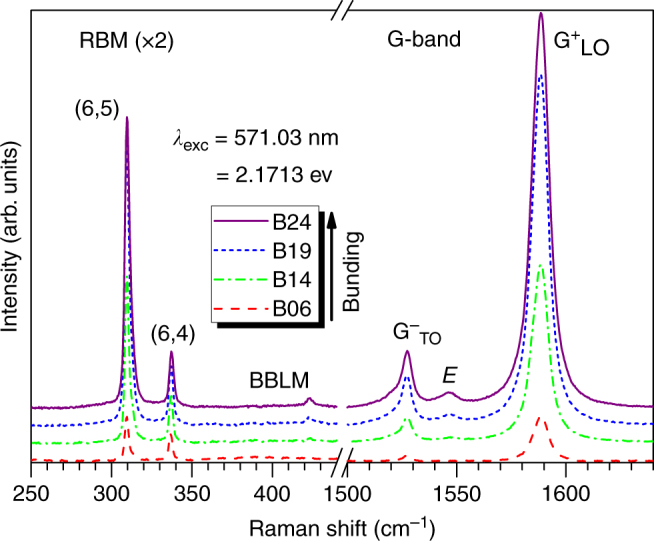
Resonance Raman spectra as a function of degree of nanotube bundling. Example Raman spectra in the RBM (×2) and G-band regions collected on $E_{22}^{\mathrm{S}}$ resonance (at ≈571 nm excitation) for the (6,5) SWCNTs. Spectra are displayed for samples in which degree of SWCNT bundling is varied from least bundled (B06) to most bundled (B24). Specific spectral features are labeled by their phonon mode designation. Curves are offset vertically for clarity

Figure 1 shows example Raman spectra of the radial breathing mode (RBM) and G-band regions taken with excitation into the second excitonic sub-band $\left( {E_{22}^{\mathrm{S}}} \right)$. In the RBM region, the (6,5) peak at 309.5 cm^−1^ dominates. The (6,5) RBM slightly hardens and broadens with bundling. In the G-band region, the longitudinal optical (LO) ${\mathrm{G}}_{{\mathrm{LO}}}^ +$ and transverse optical (TO) ${\mathrm{G}}_{{\mathrm{TO}}}^ -$ mode frequencies (≈1588 and ≈1527 cm^−1^, respectively) are relatively unaffected by bundling, though a slight broadening is observed. (RBM and G mode frequencies and linewidths may be found in Supplementary Table 2.) Symmetry breaking in the SWCNT bundles is apparent by the appearance of an *E*-symmetry mode at 1545 cm^−1^,^28,29^ which increases in intensity with bundle size. We note this feature is unlikely the TO mode of a larger-diameter SWCNT, as the corresponding RBM and absorption signatures for such a nanotube are absent^30^. Interestingly, a small peak at ≈425 cm^−1^ is also observed to appear and grow in intensity with bundle size. We tentatively assign this peak as evidence of a predicted bundle breathing-like mode (BBLM)^31^. Alternatively, it may be an *E*-symmetry mode associated with the RBM^32,33^. Finally, evidence of minor contamination by (6,4)-SWCNTs is visible as both a low-frequency shoulder^30^ on the (6,5)-${\mathrm{G}}_{{\mathrm{TO}}}^ -$ and an RBM at 336.7 cm^−1^.^19,20^ We note the observed (6,4) RBM intensity is disproportionately high with respect to its concentration. For $E_{22}^{\mathrm{S}}$ excitation, as used herein, the (6,4) scattering cross-section is significantly larger than that of the (6,5) structure due to its larger exciton–phonon coupling for this transition^19,34,35^. By taking a ratio of the (6,4) and (6,5) RBM intensities and correcting for the known difference in their exciton–phonon coupling strengths^34,35^, we estimate the (6,4) concentration to be ~(2–3)% that of the (6,5). There are no other minority (*n*, *m*) species identifiable in the excitation window of the resonant Raman spectra, which underscores the (6,5)-enriched nature of these samples.

### Raman excitation profiles

**Fig. 2:**
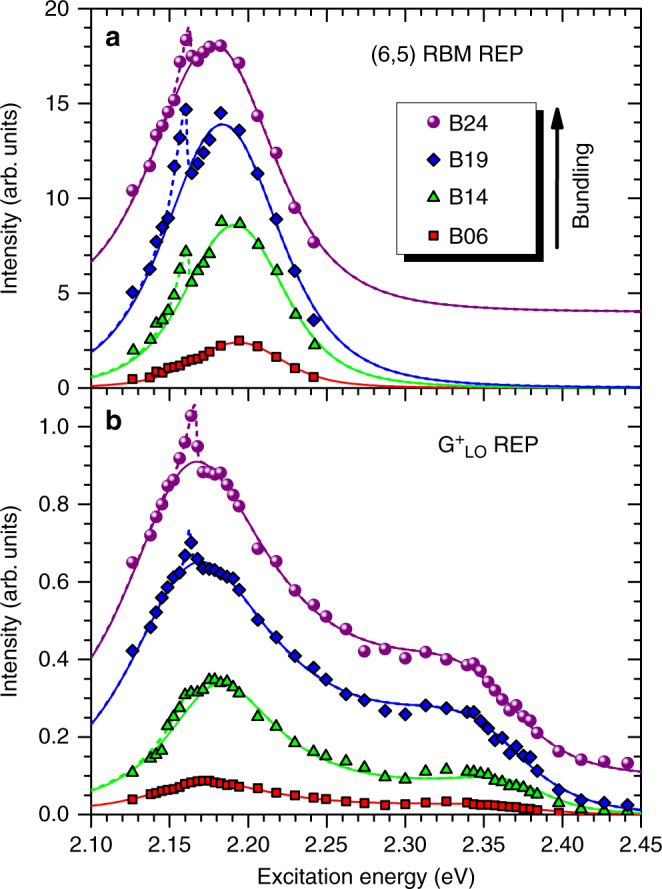
Bundle-dependent appearance of sharp anomalous resonance peak in Raman excitation profiles of (6,5)-enriched SWCNTs. REPs of the **a** RBM and **b** ${\mathrm{G}}_{{\mathrm{LO}}}^{\mathrm{ + }}$ mode with increasing bundle size for SWCNT fractions labeled B06 to B24. Data shown as symbols, fits to a Lorentzian model (as described in text) shown as lines. A sharp additional feature at ~2.16 eV appears for bundled samples and increases with bundle size. In fitting the REPs to Eq. (1), *E*_ph_ was fixed at the experimentally measured phonon frequencies. A dashed line is added over the anomalous peak (AP) feature as a guide to the eye. Curves for B24 offset vertically for clarity

The resonance Raman excitation profiles (REPs) for the (6,5) RBM, as a function of bundle size, are shown in Fig. 2a. In addition to the expected broad resonance of the $E_{22}^{\mathrm{S}}$ excitonic peak, the RBM REPs for the bundled samples include a striking new sharp feature, located just below the $E_{22}^{\mathrm{S}}$ resonance peak. Interestingly, this feature is absent from the corresponding absorption spectra. (see Supplementary Note 1) In order to more quantitatively characterize the REPs, we fit the main resonance envelope with the commonly used form

1 $${\cal I}_0 = \left| {\chi _0^{||}(\omega )} \right|^2,$$

where the Raman polarizability

2 $$\chi _0^{||} = \frac{{M_1}}{{\hbar \omega - E_{22}^{\mathrm{S}} - i{\it{\Gamma }}_{22}{\mathrm{/}}2}} + \frac{{M_2}}{{\hbar \omega - E_{22}^{\mathrm{S}} - E_{{\mathrm{ph}}} - i{\it{\Gamma }}_{22}{\mathrm{/}}2}},$$

depends on laser excitation energy, *ħω*, the exciton transition energy for the (6,5)-SWCNTs, $E_{22}^{\mathrm{S}}$, phonon energy, *E*_ph_, and associated broadening term, *Γ*_22_. The matrix elements for exciton–phonon coupling for resonance with the excitation photon (*M*_1_, incoming resonance) and with the scattered photon (*M*_2_, outgoing resonance) are taken as *M*_1_ = −*M*_2_^36^. The sharp anomalous peak (AP) is highlighted by addition of a dashed line as a guide to the eye. Table 1 gives the $E_{22}^{\mathrm{S}}$ and *Γ*_22_ values obtained from the fitting procedure. As found for the absorption spectra (see Supplementary Table 1), the REPs also indicate that transition energy and width red-shift and broaden as bundling increases. In contrast, we find the position of the RBM AP remains constant (within 1 meV, see Supplementary Table 3).

**Table 1:** Fit parameters for the (6,5) RBM and ${\mathrm{G}}_{{\mathrm{LO}}}^ +$ REPs. Bundle size decreases down the rows in the table from B24 to B06 (unbundled)

Sample	${{\bf E}}_{\bf {22}}^{{\bf S}}$ (eV)		*Γ*_22_ (meV)		*C*
	RBM	${{\bf{G}}}_{{\bf{LO}}}^ {+}$	RBM	${{\bf{G}}}_{{\bf{LO}}}^ {+}$	
B24	2.1585	2.1595	125	124	0.32
B19	2.1644	2.1610	117	115	0.28
B14	2.1724	2.1785	96	88	0.36
B06	2.1741	2.1690	84	102	0.34

The ${\mathrm{G}}_{{\mathrm{LO}}}^{\mathrm{ + }}$ REP is shown in Fig. 2b. The primary resonance response appears as two peaks. These correspond to the incoming and outgoing resonances of Eq. (1), which (unlike for the RBM) can be resolved in the case of the ${\mathrm{G}}_{{\mathrm{LO}}}^{\mathrm{ + }}$ phonon due to its frequency being sufficiently high to provide the peak separation required for resolution at the observed linewidths (84–125 meV, Table 1). We observe significant asymmetry in the ${\mathrm{G}}_{{\mathrm{LO}}}^{\mathrm{ + }}$ REP, with the outgoing resonance peak being significantly weaker than that for the incoming resonance. This effect has been observed previously in both metallic and other semiconducting SWCNTs and may be understood as an interference effect introduced by non-Condon contributions to the Raman scattering cross-section^36,37^. Most importantly, we again observe the sharp AP feature superimposed on the main resonance envelope. Additionally, we note that the AP is also apparent (and present at the same energy) in the REP of the ${\mathrm{G}}_{{\mathrm{TO}}}^ -$ mode, but is not as well-defined as in the REPs of the RBM and ${\mathrm{G}}_{{\mathrm{LO}}}^{\mathrm{ + }}$ due to poorer signal-to-noise (see Supplementary Fig. 2). We thus observe the sharp AP feature over multiple samples and for two well-separated spectral regions (associated with the different scattering wavelengths of the RBM and G phonons). While the AP increases in intensity with bundle size for the G phonon, the RBM behavior is not as clear (Fig. 2).

We model the ${\mathrm{G}}_{{\mathrm{LO}}}^{\mathrm{ + }}$ REPs in the same manner as for the RBM, while recognizing that the relation *M*_1_ = −*M*_2_ no longer holds in the case of the asymmetric response. To capture the asymmetry, a non-Condon parameter *C* = (*M*_1_ + *M*_2_)/(*M*_1_ − *M*_2_) is introduced^36,37^. We note here that, for *C* = 0, we recover *M*_1_ = −*M*_2_, as in the case of the RBM REP. In line with the RBM behavior, the ${\mathrm{G}}_{{\mathrm{LO}}}^{\mathrm{ + }}$ REPs also red-shift and broaden as bundle size increases (Table 1). Notably, the AP position again remains constant with bundle size (within 6 meV, Supplementary Table 3). Finally, we note that the *C* values obtained from the fits and listed in Table 1 show no clear trend with bundle size, but are similar in magnitude (0.2 ≤ *C* ≤ 0.4) to those obtained for other SWCNT chiralities in earlier work^36,37^.

The sharp AP that we observe has not been reported previously in mixed-chirality SWCNT bundles^20,22,23^. It is important to note that the AP is also absent in the REP for unbundled samples, yet we observe it in multiple bundled samples and in two well-separated spectral regions. It is therefore reasonable to conclude it arises due to specific intertube interactions between SWCNTs of identical chirality. In the work of Crochet et al.^14^, such interactions were shown to have the potential to generate intertube excitons, whose splitting of exciton transition energies could lead to intertube states with lowered energy in the vicinity of the observed AP. That we observe the sharp feature in the REPs for the RBM and both the ${\mathrm{G}}_{{\mathrm{LO}}}^{\mathrm{ + }}$ and ${\mathrm{G}}_{{\mathrm{TO}}}^{\mathrm{ - }}$ phonons indicates that each couples to the intertube exciton generation process. In particular, the RBM coupling, whose nuclear motions are perpendicular to the nanotube axis, is consistent with an expectation that the intertube excitons will be polarized perpendicular to the SWCNT axis.

While these spectroscopic behaviors are consistent with their origins arising from the occurrence of intertube excitons, the theory developed by Crochet et al.^14^ is insufficient to explain why their occurrence should be manifested so strongly in the altered REP. Nor does it provide a basis for understanding why the related resonance should be significantly narrower than that for the intratube excitons. In the following section, we develop a model accounting for the intratube and intertube exciton scattering that reproduces the relevant experimental observations and strengthens our assignment of the sharp resonance arising as a consequence of intertube exciton generation. Furthermore, the model holds additional implications for understanding intertube excitons in chirally-defined SWCNT bundles and their relation to interlayer excitons in 2D systems.

### Theoretical model

**Fig. 3:**
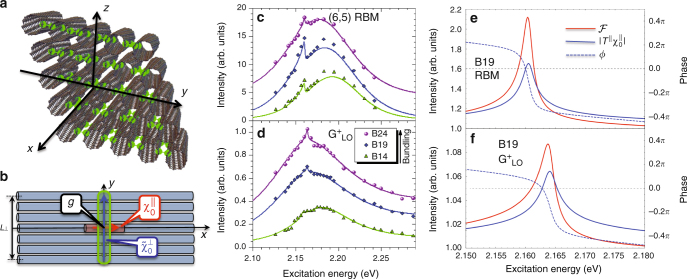
Model for intertube exciton generation in (6,5)-SWCNT bundles and origin of Fano resonance via coupling of intertube and intratube excitons. **a** Optimized (6,5)-SWCNT bundle structure obtained via molecular dynamics simulations. Atomic sites of adjacent tubes separated by van der Waals distance *d*_0_ ≤ 3.14 Å are marked in green. These sites participate in the intertube electron/hole tunneling facilitating formation of the intertube exciton state. **b** The *xy*-plane cross-section of the bundle. Red cylinder marks the intratube exciton state polarized along the tube axes as indicated by the red arrow. It gives rise to the Raman polarizability $\chi _0^{||}(\omega )$. Green area marks an intertube exciton state delocalized across *L*_⊥_ tubes and polarized (blue arrow) across the bundle. This state results in the Raman polarizability $\tilde \chi _0^ \bot (\omega )$. Local coupling, *g*, between the two types of excitons at the intercept point gives rise to their scattering. Fano resonance appears as a signature of the bright intratube exciton scattering by the dark intertube exciton. **c**, **d** Experimental data fit (solid lines) using our theoretical model [Eqs. (5) and (6)]. **e**, **f** Fano lineshape function (red), the absolute values of the scattering term (blue solid), and the scattering phase (blue dash) calculated using parameters obtained from experimental data fit for the B19 bundle RBM and ${\mathrm{G}}_{{\mathrm{LO}}}^{\mathrm{ + }}$ modes, respectively. Positive (negative) phase values define spectral regions of constructive (destructive) interference

In ref.^14^, correlated tunneling of an electron and hole results in the formation of an intertube exciton state. The process was shown to weakly perturb $E_{11}^{\mathrm{S}}$ exciton energies, resulting in only minor tunneling splitting and an intertube exciton state with energy close to the intratube state^14^. Here we assume a similar process, with weak energy splitting, is valid for the $E_{22}^{\mathrm{S}}$ exciton state probed *via* the REPs. An optimized (6,5)-SWCNT bundle structure is obtained via molecular dynamics simulations (Fig. 3a). Atomic sites between adjacent SWCNTs that are separated by van der Waals distance *d*_0_ = 3.14 Å or less are identified as those that facilitate the intertube exciton tunneling and are marked in green. It is clear from the plot that these sites effectively form 1D chains across the bundle, suggesting a means for 1D delocalization of the intertube excitons. As we further illustrate in Fig. 3b, the intertube and intratube exciton states can locally interact with energy *g*, which results in their scattering at the interaction sites.

Using the exciton scattering model developed in Supplementary Note 2, we evaluated the Raman polarizability tensor for the interacting exciton states. The tensor consists of two diagonal (intra- and intertube) components and one off-diagonal cross term. The intertube exciton state, however, is expected to have a weak direct contribution to the Raman tensor and is therefore assumed to be dark. This is a reasonable expectation in comparison to other transitions polarized perpendicular to the nanotube axis. For example, the so-called cross-polarized transitions (*E*_12_ and *E*_21_) are significantly weaker than their parallel polarized counterparts ($E_{11}^{\mathrm{S}}$ and $E_{22}^{\mathrm{S}}$)^38–40^. Therefore, the leading contribution to the Raman response comes from the following tensor component polarized along the bundle

3 $$\alpha ^{||}(\omega ) = \frac{{\chi _0^{||}(\omega )}}{{1 - g^2\tilde \chi _0^ \bot (\omega )\chi _0^{||}(\omega )}}.$$

Here, the noninteracting intratube Raman polarizability $\chi _0^{||}(\omega )$ has the experimentally validated form given by Eq. (2). The non-interacting intertube exciton response can be approximated as (see Supplementary Note 2 for details)

4 $$\tilde \chi _0^ \bot (\omega ) = \frac{{M_0}}{{\sqrt {\beta \left( {\hbar \omega - E_0 - i{\it{\Gamma }}_0{\mathrm{/}}2} \right)} }},$$

where *ħω* is the laser excitation energy, *E*_0_ is the intertube exciton band minimum and *Γ*_0_ is the associated linewidth. *M*_0_ is a complex quantity combining the matrix elements for exciton-photon and exciton–phonon coupling. *β* is the intertube exciton tunneling energy normalized per $L_ \bot ^2$. Notice that according to Eq. (3) in the case of uncoupled intertube and intratube excitons, i.e., *g* = 0, the Raman polarizability identically reproduces the non-interacting exciton Raman response, i.e., $\alpha ^{||}(\omega ) = \chi _0^{||}(\omega )$. Below, we consider a regime in which the coupling parameter, *g*, is strong enough to compensate the weak values of *M*_0_ in $\tilde \chi _0^ \bot (\omega )$, which otherwise makes no contribution to Eq. (3).

We first note, however, that coupling of the intertube and intratube excitons introduces an interference between two pathways contributing to the REP intensity: first is the non-perturbed intratube exciton response described by $\chi _0^{||}(\omega )$, and the other reflects the scattering of the intratube exciton by the intertube exciton, which affects the phase of the scattered wave. Partitioning of the response function into these two pathways can be demonstrated through a scattering matrix formalism (Supplementary Notes 2, 5, and discussion below). Importantly, this interference, originating in the exciton coupling, gives rise to a Fano resonance in the scattering response.

To examine spectroscopic signatures of the coupled exciton dynamics, we use Eq. (3) to evaluate the REP intensity that is proportional to its absolute value^41^. Neglecting numerical pre-factors, we obtain (see Supplementary Note 3 for details)

5 $${\cal I}(\omega ) = {\cal I}_0(\omega ){\cal F}(\omega ),$$

where the background contribution, ${\cal I}_0(\omega )$, is given by Eqs. (1) and (2). The lineshape function

6 $${\cal F} = \frac{{\left( {\epsilon + q} \right)^2 + \gamma _0^2}}{{\epsilon ^2 + 1}},$$

describes a generalized Fano profile. Its parameters are related to the parameters entering the intratube and intertube exciton polarizabilities [Eqs. (2) and (4)]. Specifically, the term determining the energy detuning from the Fano resonance is $\epsilon = {\mathrm{Re}}\left\{ {\sqrt {\beta \left( {\hbar \omega - E_0 - i{\it{\Gamma }}_0{\mathrm{/}}2} \right)} - g^2M_0\chi _0^{||}(\omega )} \right\}{\mathrm{/}}\gamma$, the net damping rate is $\gamma = {\mathrm{Im}}\left\{ {g^2M_0\chi _0^{||}(\omega ) - \sqrt {\beta \left( {\hbar \omega - E_0 - i{\it{\Gamma }}_0{\mathrm{/}}2} \right)} } \right\}$, and the spectrum asymmetry is $q = g^2{\mathrm{Re}}\left\{ {M_0\chi _0^{||}(\omega )} \right\}{\mathrm{/}}\gamma$.

The ratio $\gamma _0 = {\mathrm{Im}}\sqrt {\beta \left( {\hbar \omega - E_0 - i{\Gamma} _0{\mathrm{/}}2} \right)} {\mathrm{/}}\gamma$ accounts for the finite line widths of the intertube exciton states. In many cases, it is assumed that *Γ*_0_ = 0, resulting in *γ*_0_ = 0, which recovers the conventional form of the Fano linshape function^42,43^. However, the intertube exciton has finite line width, *Γ*_0_, which has to be accounted for using a generalized expression (6).

As applied to the experimental results, this model provides an excellent fit to the data for both the RBM (Fig. 3c) and G mode (Fig. 3d) REPs (see Supplementary Note 4 for details and Supplementary Table 3 for fitting parameters). In Fig. 3e,f, we also show the associated Fano contribution to the overall REP for the B19 bundle batch (all Fano profiles are presented in Supplementary Fig. 3). Each resonance has a sharp asymmetric peak formed as a result of constructive and destructive interference between coupled intertube and intratube exciton states. To demonstrate this, we represent the Fano lineshape function as (see Supplementary Note 5 for details)

7 $${\cal F}(\omega ) = \left| {1 + \left| {T^{||}(\omega )\chi _0^{||}(\omega )} \right|e^{i\phi (\omega )}} \right|^2,$$

where the first (unity) term is due to the intratube exciton response only and the second one describes the scattering of the intratube exciton by the intertube exciton with the scattering matrix element $T^{||}(\omega ) = g^2\chi _0^ \bot (\omega ){\mathrm{/}}\left[ {1 - g^2\tilde \chi _0^ \bot (\omega )\chi _0^{||}(\omega )} \right]$. Although the absolute value of the scattering term squared, $\left| {T^{||}(\omega )\chi _0^{||}(\omega )} \right|^2$, contributing to ${\cal F}(\omega )$ has symmetric quasi-Lorentzian form, the scattering induced phase *ϕ*(*ω*) variation through zero in the interference term, $2\left| {T^{||}(\omega )\chi _0^{||}(\omega )} \right|{\mathrm{cos}}{\kern 1pt} \phi (\omega )$, changes its sign and subsequently gives rise to the sharp asymmetry of the total lineshape. Panels e and f of Fig. 3 illustrate this situation.

## Discussion

The strength of the Fano resonance is determined by the product of the coupling strengths *g*^2^ and the matrix elements *M*_0_ and *M*_1_ representing a combination of the associated exciton oscillator strengths and the exciton–phonon couplings. In our case, for which a direct contribution to the Raman response by the intertube exciton is minimized due to its weak oscillator strength (i.e., the intertube exciton is dark), its presence is still manifested by the Fano resonance, whose intensity is enhanced by the coupling strength *g*^2^ and by the exciton–phonon terms entering the *M*_0_*M*_1_ product. The total REP results from the product of the broad background spectrum due to uncoupled intratube excitons and the Fano contribution. Accordingly, the sharp asymmetry on the high-energy side of the experimental AP is seen in panels c and d of Fig. 3 to arise directly from the Fano contribution and is a direct signature of the intertube and intratube exciton coupling. On the other hand, the absorption cross-section does not depend on the exciton–phonon interaction. As a result, the contribution of the intertube exciton does not receive enough enhancement to be observed in the absorption spectra as indicated by the experiment.

Our theory, thus, provides a basis for understanding the presence of the AP as a signature of the intertube exciton formation. The latter is very sensitive to the bundle structure. To ensure generation of a sufficiently large population of the intertube exciton states (i.e., the 1D chains shown in Fig. 3a), the SWCNTs within the bundle should form a hexagonal structure. This explains the absence of the AP in earlier studies of SWCNT bundles of mixed chirality^20,22,23^. The bundles used in this study have high purity and are assumed to form such hexagonal structures with low levels of imperfections (likely present to some degree as a result of minor concentration of (6,4) structures, see Fig. 1). Furthermore, an increase in bundle size should result in a larger population of the intertube excitons appearing as an enhancement of the AP intensity with bundle size. This expectation is supported by the G-mode REPs, but is not as clear a trend for the RBM data (Fig. 2a, b). Additionally, it is likely that in sufficiently large bundles the outer shell of nanotubes may stabilize the internal hexagonal structure by protecting it from environmental fluctuations. This should further contribute to the enhancement of the AP intensity.

Recently, Fano interference has received broad attention in photonics including nano-plasmonics and meta-materials studies, in which a broad super-radiant resonant mode interferes with a narrow dark mode, resulting in Fano features in light scattering signals^44,45^. The analogy between our model and models used in nano-plasmonics becomes even deeper in light of the classical photon scattering model proposed in ref.^46^, which is conceptually similar to our quantum mechanical exciton scattering model. In the same article, the authors also draw a basic analogy between the complex nature of generalized Fano resonances in the latter model and appearance of the generalized Fano lineshape [Eq. (6)] in the response of two linearly coupled damped harmonic oscillators, one of each being bright with broad spectral response, while the other is dark and has a narrow line overlapping with the bright oscillator line. (The same analogy is extensively used in a review article^45^.) In Supplementary Note 6, we demonstrate that by assuming the dark (the bright) intertube (intratube) exciton state has a response function of a classical oscillator with narrow (broad) linewidth, we can exactly reproduce the Fano response obtained in ref.^46^. This shows that details of the dark and bright exciton structures do not affect the appearance of the Fano resonance but rather influence details of the linshape.

This universality suggests that Fano resonances can be expected to be observed in the REP of 2D layered materials, assuming interacting intralayer and interlayer exciton states, in which a dark interlayer exciton state has a narrow resonance that is well coupled to, and overlapping with, the broad resonance of the bright intralayer excitons. Furthermore, the simple oscillator model disregards the effect of the interlayer exciton delocalization, suggesting that the Fano resonance can appear even in bi-layered systems, provided the coupling between different types of excitons is strong enough and the population of the interlayer excitons is high. The latter can be favored in 2D geometry by a large overlap area that thus increases the number of tunneling sites. In addition, the sensitivity of the Fano lineshape on the scattering phase can be utilized to reveal additional microscopic information on complex exciton interactions.

As processes for isolating specific nanotube chiralities mature and interest grows in the behavior and applications of interacting assemblies of SWCNTs enriched in a single chirality (such as in photovoltaic assemblies and in optoelectronic devices)^16^, understanding the nature of intertube interactions in compositionally-defined aggregates becomes increasingly important. In addition to observing the expected red-shifting and broadening of electronic transitions in small bundles enriched in (6,5) SWCNTs, our REP studies have revealed the appearance of an unexpected prominent sharp feature superimposed on the intrinsic Raman excitation response. We have demonstrated that this new spectroscopic feature is a signature of the presence of intertube excitons whose generation is enhanced by the unique geometric structure arising in enriched-composition bundles.

Importantly, applying our model for this behavior to fitting of our experimental data indicates that the sharp REP feature arises not as a direct response of the uncoupled intertube exciton itself, but instead is due to scattering arising from the interaction between intertube and intratube excitons. The result is a Fano resonance that defines the unique lineshape we observe: a Lorentzian-like rise to the peak intensity on the low-energy side, associated with the constructive interference, paired with a sharp decay in intensity on the high-energy side, as a signature of the destructive interference. Our generalized description links the behavior observed in SWCNTs to plasmonic and meta-materials responses of increasing importance in nanophotonics and may be extended to implications for 2D materials and SWCNT defect states.

## Methods

### SWCNT chirality enrichment and bundling

SWCNT suspensions in aqueous 2% by weight sodium cholate were prepared from CoMoCat SG65 (Southwest Nanotechnologies) starting material and enriched in the (6,5) structure using density gradient ultracentrifugation (DGU), as described previously^14,47^. Aggregation and fractionation of variably-sized -enriched bundles were then generated exactly as described before^14^. Briefly, (6,5)-enriched material was dialysed with deionized water to induce SWCNT aggregation. DGU was used to fractionate aggregates by bundle size. Progressively larger bundles appear as fractions (extracted as 150 μL volumes) of increasing buoyant density. Specific fractions used in this study are labeled in order of increasing bundle size as B06 (unbundled), B14, B19, and B24. AFM characterization of bundle size appears in earlier work^14^ and absorption spectra (Supplementary Fig. 1) for each fraction were recorded on a Cary 6000i UV-Vis-near-IR spectrometer.

Certain equipment, instruments, or materials are identified in this paper in order to adequately specify the experimental details. Such identification does not imply recommendation by the National Institute of Standards and Technology nor does it imply the materials are necessarily the best available for the purpose.

### Resonance Raman spectroscopy

Resonance Raman scattering was performed on a JY-Horiba Dilor XY 800 triple-grating Raman spectrometer with a liquid nitrogen cooled CCD detector in 180° backscattering geometry. A Coherent 599–21 dye laser pumped with a Coherent Sabre Ar ion laser provides tunable excitation over the spectral range from 500 to 585 nm using Coumarin 521 and Rhodamine 110 laser dyes. Laser power remained approximately constant and below 30 mW in a sample spot size of ≲80 μm. Spectra were obtained from aqueous SWCNT samples and Raman reference standards inserted into glass NMR tubes. Benzonitrile peaks at 460.9 and 1598.9 cm^−1^ serve as frequency^48^ and scattering intensity^49,50^ calibrants for the radial breathing mode (RBM) and G-band regions, respectively.

### Data availability

The data that support the findings of this study are available from the authors on reasonable request.

## Electronic supplementary material

Supplementary Information
