# Supplementary material for: Resonance Raman signature of intertube excitons in compositionally-defined carbon nanotube bundles
Source: Nat Commun. 2018 Feb 12;9:637. doi: 10.1038/s41467-018-03057-7 (PMC5809379; doi:10.1038/s41467-018-03057-7)
Supplement: Supplementary file 1 — Supplementary Information [file 41467_2018_3057_MOESM1_ESM.pdf]

Supplementary Information: Resonance Raman Signature of Intertube Excitons in Compositionally-Defined Carbon Nanotube Bundles

Jeffrey R. Simpson,[1,2] Oleksiy Roslyak,[3] Juan G. Duque,[4] Erik H. Hároz,[5] Jared J. Crochet,[4] Hagen Telg,[5] Andrei Piryatinski,[6] Angela R. Hight Walker,[1] and Stephen K. Doorn[5]

[1]Engineering Physics Division, National Institute of Standards and Technology (NIST), Gaithersburg, MD 20899, USA

[2]Department of Physics, Astronomy, and Geosciences, Towson University, Towson, MD 21252, USA

[3]Physics and Engineering Physics, Fordham University, Bronx, New York 10458, USA

[4]Chemistry Division, Physical Chemistry and Applied Spectroscopy, Los Alamos National Laboratory, Los Alamos, New Mexico 87545, USA

[5]Center for Integrated Nanotechnologies, Los Alamos National Laboratory, Los Alamos, New Mexico 87545, USA

[6]Theoretical Division, Los Alamos National Laboratory, Los Alamos, New Mexico 87545, USA

Supplementary Note 1: UV-vis-NIR absorption.

Supplementary Figure 1 compares the optical absorption spectra in the near-infrared to ultraviolet spectral region for three samples of increasing bundle size: B06 (unbundled), B14, and B19. Regions containing the E_{11}^{S} and E_{22}^{S} intratube excitonic transitions, specifically those for $(6,5)$, are indicated. The excitonic peaks redshift and broaden with increasing bundle size. Additionally, spectral weight from both the E_{11}^{S} and E_{22}^{S} features redistributes into the non-excitonic absorption background with bundling. These results are consistent with earlier reports[1–4] on bundled SWCNTs and provide spectroscopic evidence supporting the increase of bundle size with buoyant density.[3] Additional, weak absorption peaks include minority (n,m) species and phonon sidebands, identified as follows, a: E_{11}^{S} of $(9,1)$, b: E_{11}^{S} of $(6,4)$, c: K-point phonon plus E_{11}^{S} of $(6,5)$,[5] d: E_{22}^{S} of $(6,4)$, e: K-point phonon plus E_{22}^{S} of $(6,5)$,[5] and f: metal of unidentified (n,m). No new absorption peaks are observed with increasing bundle size. The $(6,5)$ E_{11}^{S} and E_{22}^{S} energies and line widths are tabulated in Supplemental Table 1. The shaded gray region in Fig. 1 indicates the $(6,5)$-E_{22}^{S} excitation range used for resonant Raman spectroscopy.

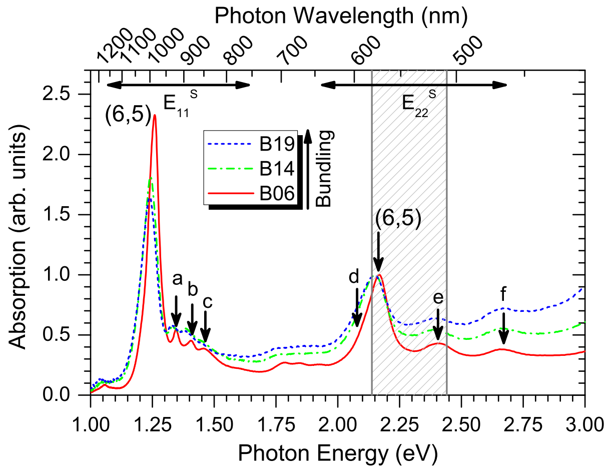

Supplementary Figure 1: **Absorption spectra in the near-infrared (NIR) to ultraviolet (UV) energy range for three fractions.** The $(6,5)$ E_{11}^{S} and E_{22}^{2} excitonic transitions are labeled. As bundling increases, the absorption peaks redshift and broaden. The gray shaded region indicates the range of excitation for resonance Raman measurements. Additional absorption peaks include minority (n,m) species and phonon sidebands, identified as follows – a: $(9,1)$ E_{11}^{S}, b: $(6,4)$ E_{11}^{S}, c: K-point phonon for $(6,5)$,[5] d: $(6,4)$ E_{22}^{S}, e: K-point phonon for $(6,5)$,[5] and f: metal of unidentified (n,m).

Supplementary Table 1: UV-vis parameters for $(6,5)$ SWCNTs.

Sample	E_{11}^{S} (eV)	$\mathit{\Gamma}_{11}$ (meV)	E_{22}^{S} (eV)	$\mathit{\Gamma}_{22}$ (meV)
B19	1.243	62	2.157	109
B14	1.246	60	2.159	108
B06	1.258	47	2.172	87

Supplementary Table 2: **Raman spectra: Phonon energies and widths.** Spectral fitting analysis of the Raman-active RBM and G_{LO}^{+} phonon modes for $(6,5)$ bundled SWCNTs. Bundle size decreases down rows in the table from B24 to B06 (unbundled).

Sample	ω_{RBM} (cm^{-1})	$\mathit{\Gamma}_{RBM}$ (cm^{-1})	ω_{G^+} (cm^{-1})	$\mathit{\Gamma}_{G^+}$ (cm^{-1})
B24	310.0	3.5	1588.4	9.3
B19	309.7	3.2	1588.3	9.0
B14	309.5	2.6	1588.3	9.1
B06	309.5	2.6	1588.3	8.0

Supplementary Table 3: **Data fit with Fano lineshape:** Fit parameters for the $(6,5)$ RBM and G_{LO}^{+} REPs for bundled SWCNTs. Bundle size decreases down the rows in the table from B24 to B14.

Sample	E_0 (eV)		$\mathit{\Gamma}_0$ (meV)		φ_0		M ($\times 10^{-4}$)	
	RBM	G_{LO}^{+}	RBM	G_{LO}^{+}	RBM	G_{LO}^{+}	RBM	G_{LO}^{+}
B24	2.1606	2.1639	2.0	2.0	4.3883	3.793	2.47	1.32
B19	2.1609	2.1641	2.0	2.0	3.6995	3.518	6.38	0.87
B14	2.1609	2.1586	2.0	3.4	3.2898	3.608	6.28	1.82

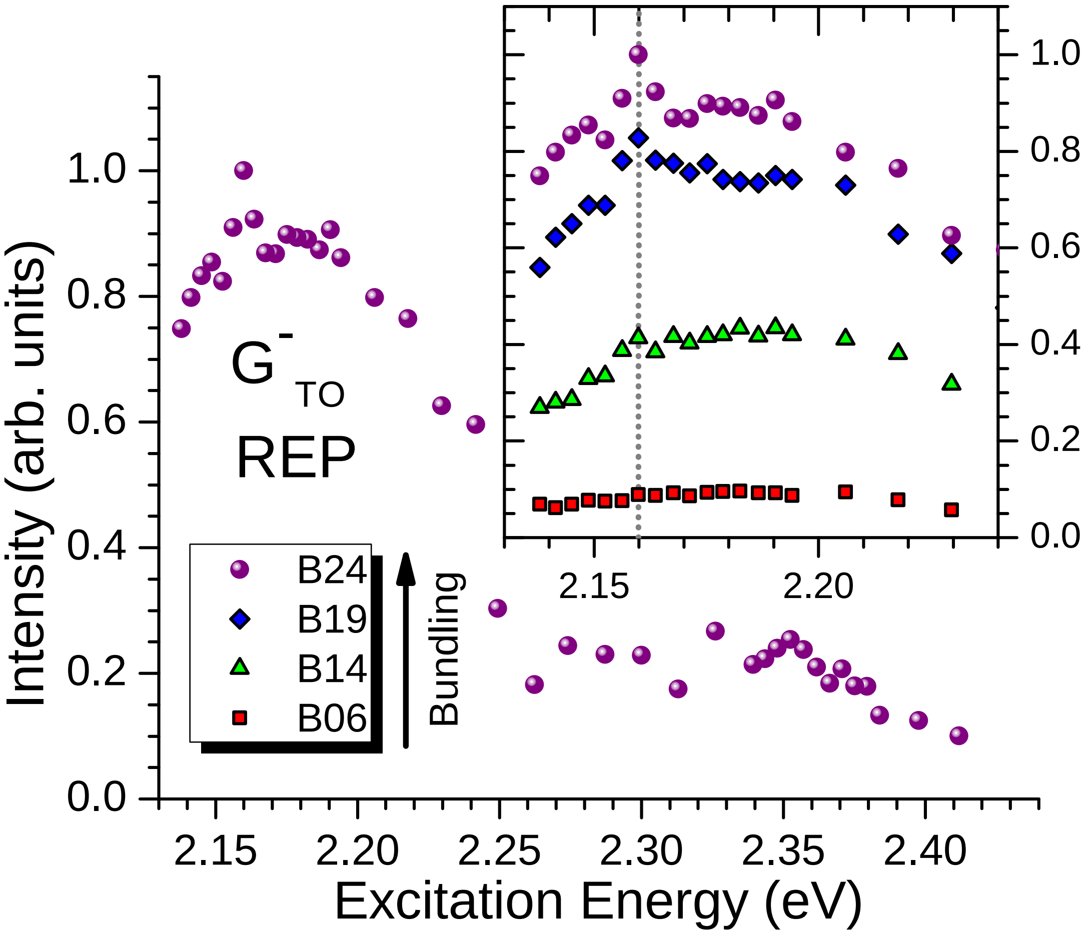

Supplementary Figure 2: Full resonance excitation profile (REP) for the G^{-}_{TO} mode of the most bundled sample (B24, main figure) and partial REPs (inset) centered around the incoming resonance peak for the G^{-}_{TO} mode with increasing bundle size for SWCNT fractions B06 (unbundled) to B24. Decreased signal-to-noise in the Raman scattering intensity for the weaker G^{-}_{TO} mode results in a less well-defined anomalous peak feature. Nevertheless, the anomalous peak is still apparent and occurs at approximately the same excitation energy (2.16 eV as highlighted by the vertical dashed line in the inset) as observed for the RBM and G^{+}_{LO} REPs. We also note that the asymmetric response of the outgoing resonance remains apparent in the G^{-}_{TO} mode of the full REP for B24, as previously reported.[6]

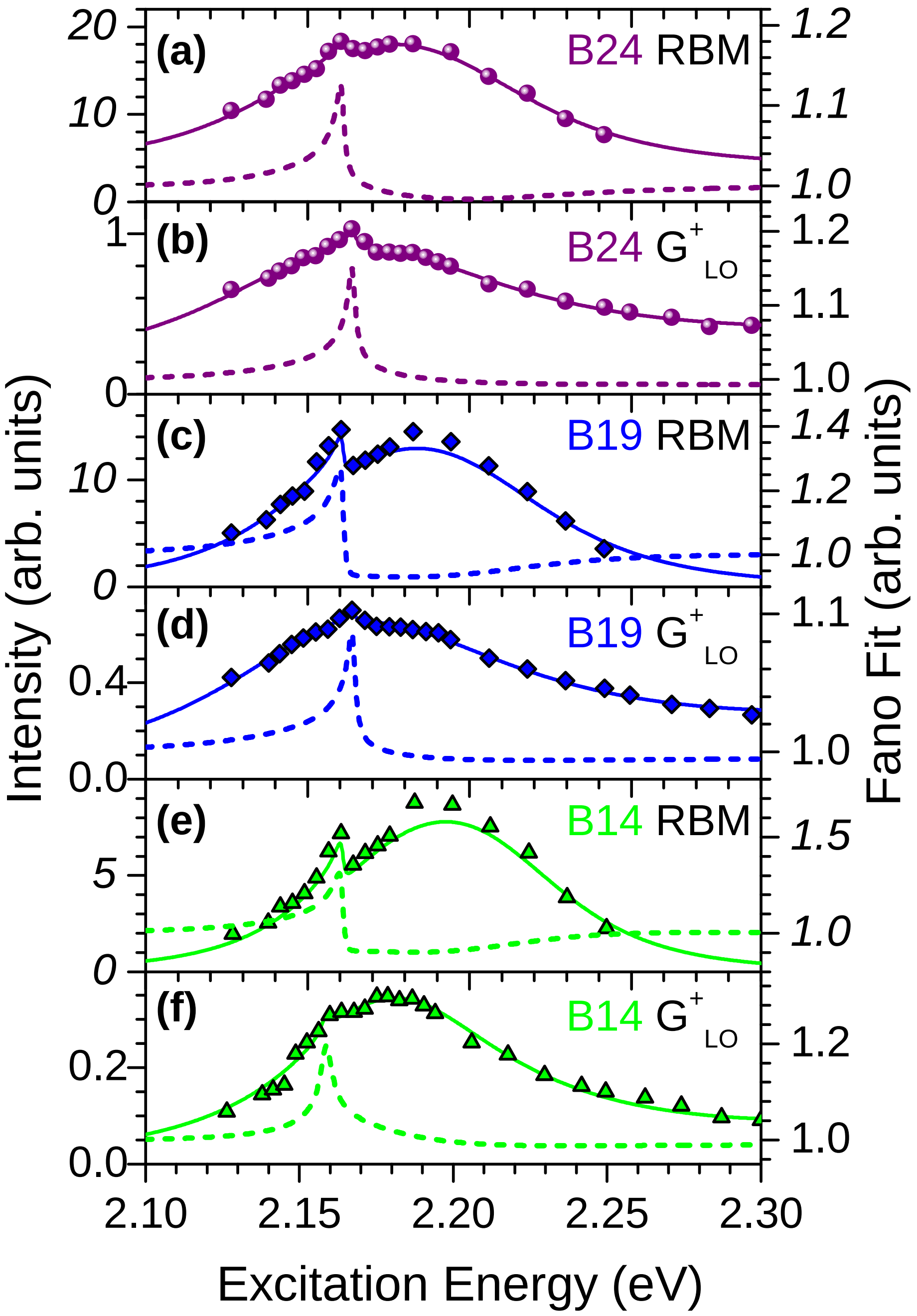

Supplementary Figure 3: Resonance excitation profiles (REPs) of the RBM (a), (c), and (e) and G^{+}_{LO} (b), (d), and (f) for the bundled SWCNTs. Data are shown as symbols along with the full intensity fit shown as a solid line. The Fano lineshapes (dashed curves) are plotted on the secondary vertical axes.

Supplementary Note 2: Exciton scattering model and Raman polarizability.

Let us introduce a quantity $\chi^{\perp}_{|n-m|}$ which is the coordinate space electronic polarizability associated with a 1D exciton state delocalized across $L_{\perp}$ sites (tubes) ($i.e.$, $n, m = 1, \ldots L_{\perp}$) of a bundle as illustrated in Fig. 3 (a) and (b) in the main text. This delocalized *intertube* exciton overlaps with localized *intratube* states at site n_0. Further, introducing the local electronic polarizability, $\chi^{\parallel}_0$, for the intratube exciton, we present the following two-component exciton polarizability tensor associated with the noninteracting inter- and intratube excitons

$$\overset{\leftrightarrow}{\chi}{}^{0}_{nm} = \overset{\leftrightarrow}{\chi}{}^{\perp}_{|n-m|} + \overset{\leftrightarrow}{\chi}{}^{\parallel}_{0}\delta_{n_0,n}\delta_{n_0,m}. \tag{1}$$

Here,

$$\overset{\leftrightarrow}{\chi}{}^{\perp}_{|n-m|} = \begin{pmatrix} \chi^{\perp}_{|n-m|} & 0 \\ 0 & 0 \end{pmatrix}, \tag{2}$$

and

$$\overset{\leftrightarrow}{\chi}{}^{\parallel}_{0} = \begin{pmatrix} 0 & 0 \\ 0 & \chi^{\parallel}_{0} \end{pmatrix}, \tag{3}$$

are the intertube and intratube polarizability matrices, respectively. For the sake of brevity, the dependance of the exciton polarizabilities on the frequency, ω, and phonon coordinates is implicit.

Being proportional to the associated exciton (electronic) Green's function, the total polarizability, $\overset{\leftrightarrow}{\chi}_{nm}$, of interacting inter- and intratube excitons obeys the following Dyson equation

$$\overset{\leftrightarrow}{\chi}_{nm} = \overset{\leftrightarrow}{\chi}{}^{0}_{nm} + \sum_{rs} \overset{\leftrightarrow}{\chi}{}^{0}_{nr} \overset{\leftrightarrow}{V}_{rs} \overset{\leftrightarrow}{\chi}_{sm}, \tag{4}$$

where the local (on-site) scattering potential term

$$\overset{\leftrightarrow}{V}_{nm} = \overset{\leftrightarrow}{V}\delta_{n_0,n}\delta_{n_0,m}, \tag{5}$$

is off-diagonal

$$\overset{\leftrightarrow}{V} = \begin{pmatrix} 0 & v \\ v & 0 \end{pmatrix}, \tag{6}$$

with v being the interaction energy.

We further represent the Dyson equation (Supplementary Equation (4)) in the k-space

$$\tilde{\overleftrightarrow{\chi}}_{kk'} = \tilde{\overleftrightarrow{\chi}}^{0}_{kk'} + \frac{1}{L_\perp^2}\sum_{qq'} \tilde{\overleftrightarrow{\chi}}^{0}_{kq}\tilde{\overleftrightarrow{V}}_{qq'}\tilde{\overleftrightarrow{\chi}}_{q'k'}, \tag{7}$$

where the Fourier transformed matrix elements are

$$\tilde{\overleftrightarrow{\chi}}_{kk'} = \sum_{nm} \overleftrightarrow{\chi}_{nm} e^{-ikn+ik'm}, \tag{8}$$

$$\tilde{\overleftrightarrow{\chi}}^{0}_{kq} = \sum_{nm} \overleftrightarrow{\chi}^{0}_{nm} e^{-ikn+iqm}, \tag{9}$$

$$\tilde{\overleftrightarrow{V}}_{qq'} = \sum_{nm} \overleftrightarrow{V}_{nm} e^{-iqn+iq'm}. \tag{10}$$

Specifically for the the noninteracting exciton polarizability, we obtain

$$\tilde{\overleftrightarrow{\chi}}^{0}_{kq} = L_\perp \delta_{k,q}\ \tilde{\overleftrightarrow{\chi}}^{\perp}_{k} + \overleftrightarrow{\chi}^{\|}_{0} e^{-i(k-q)n_0}, \tag{11}$$

where

$$\tilde{\overleftrightarrow{\chi}}^{\perp}_{k} = \begin{pmatrix} \tilde{\chi}^{\perp}_{k} & 0 \\ 0 & 0 \end{pmatrix}, \tag{12}$$

and $\overleftrightarrow{\chi}^{\|}_{0}$ is given by Supplementary Equation (3). Finally, the Fourier transformed scattering potential reads

$$\tilde{\overleftrightarrow{V}}_{qq'} = \overleftrightarrow{V} e^{-(q-q')n_0}, \tag{13}$$

with $\overleftrightarrow{V}$ given by Supplementary Equation (6). Substitution of Supplementary Equations (11) and (13) into Supplementary Equation (7) results in

$$\tilde{\overleftrightarrow{\chi}}_{kk'} = L_\perp \delta_{k,k'}\ \tilde{\overleftrightarrow{\chi}}^{\perp}_{k} + \overleftrightarrow{\chi}^{\|}_{0} e^{-i(k-k')n_0} + \frac{1}{L_\perp}\sum_{q} e^{-ikn_0}\left(\tilde{\overleftrightarrow{\chi}}^{\perp}_{k} + \overleftrightarrow{\chi}^{\|}_{0}\right)\overleftrightarrow{V}\tilde{\overleftrightarrow{\chi}}_{qk'} e^{iqn_0}. \tag{14}$$

To solve Supplementary Equation (14), we introduce an auxiliary function

$$\overleftrightarrow{f}_{k'}(n_0) = \frac{1}{L_\perp}\sum_{q} \tilde{\overleftrightarrow{\chi}}_{qk'} e^{iqn_0}, \tag{15}$$

in terms of which Supplementary Equation (14) becomes

$$\tilde{\overleftrightarrow{\chi}}_{kk'} = L_\perp \delta_{k,k'}\ \tilde{\overleftrightarrow{\chi}}^{\perp}_{k} + \overleftrightarrow{\chi}^{\|}_{0} e^{-i(k-k')n_0} + e^{-ikn_0}\left(\tilde{\overleftrightarrow{\chi}}^{\perp}_{k} + \overleftrightarrow{\chi}^{\|}_{0}\right)\overleftrightarrow{V}\overleftrightarrow{f}_{k'}(n_0). \tag{16}$$

To close, we multiply both sides of Supplementary Equation (16) by e^{ikn_0} and sum over k. This results in

$$\overset{\leftrightarrow}{f}_{k'}(n_0) = \left(\overset{\leftrightarrow}{\tilde{\chi}}{}^{\perp}_{k'} + \overset{\leftrightarrow}{\chi}{}^{\|}_{0}\right) e^{ik'n_0} + \left(\overset{\leftrightarrow}{\chi}{}^{\perp}_{0} + \overset{\leftrightarrow}{\chi}{}^{\|}_{0}\right) \overset{\leftrightarrow}{V} \overset{\leftrightarrow}{f}_{k'}(n_0), \tag{17}$$

where

$$\overset{\leftrightarrow}{\chi}{}^{\perp}_{0} = \frac{1}{L_\perp} \sum_k \overset{\leftrightarrow}{\tilde{\chi}}{}^{\perp}_{k}, \tag{18}$$

denotes the real space $\overset{\leftrightarrow}{\chi}{}^{\perp}_{|n-m|}$ at $n = m$.

Solution of Supplementary Equation (17) can be written in the following matrix form

$$\overset{\leftrightarrow}{f}_{k'}(n_0) = \left[\overset{\leftrightarrow}{I} - \left(\overset{\leftrightarrow}{\chi}{}^{\perp}_{0} + \overset{\leftrightarrow}{\chi}{}^{\|}_{0}\right) \overset{\leftrightarrow}{V}\right]^{-1} \left(\overset{\leftrightarrow}{\tilde{\chi}}{}^{\perp}_{k'} + \overset{\leftrightarrow}{\chi}{}^{\|}_{0}\right) e^{ik'n_0}. \tag{19}$$

Its subsequent substitution into Supplementary Equation (16) provides the solution of the Dyson equation in the form

$$\overset{\leftrightarrow}{\tilde{\chi}}_{kk'} = L_\perp \delta_{k,k'} \overset{\leftrightarrow}{\tilde{\chi}}{}^{\perp}_{k} + \overset{\leftrightarrow}{\chi}{}^{\|}_{0} e^{-i(k-k')n_0} + \left(\overset{\leftrightarrow}{\tilde{\chi}}{}^{\perp}_{k} + \overset{\leftrightarrow}{\chi}{}^{\|}_{0}\right) \overset{\leftrightarrow}{T} \left(\overset{\leftrightarrow}{\tilde{\chi}}{}^{\perp}_{k'} + \overset{\leftrightarrow}{\chi}{}^{\|}_{0}\right) e^{-i(k-k')n_0}, \tag{20}$$

containing scattering operator

$$\overset{\leftrightarrow}{T} = \overset{\leftrightarrow}{V} \left[\overset{\leftrightarrow}{I} - \left(\overset{\leftrightarrow}{\chi}{}^{\|}_{0} + \overset{\leftrightarrow}{\chi}{}^{\perp}_{0}\right) \overset{\leftrightarrow}{V}\right]^{-1}. \tag{21}$$

Substitution of Supplementary Equations (2) (with $n = m$), (3), and (6), into Supplementary Equation (21), results in the following matrix representation for the scattering operator

$$\overset{\leftrightarrow}{T} = \frac{v}{1 - v^2 \chi_0^\perp \chi_0^\|} \begin{pmatrix} v\chi_0^\| & 1 \\ 1 & v\chi_0^\perp \end{pmatrix}. \tag{22}$$

Further inserting Supplementary Equation (22) into Supplementary Equation (20) along with Supplementary Equations (2) where we set $n = m$, and Supplementary Equations (3), (12) and (22), we obtain the matrix representation for the exciton polarizability

$$\overset{\leftrightarrow}{\tilde{\chi}}_{kk'} = \begin{pmatrix} L_\perp \delta_{k,k'} \tilde{\chi}_k^\perp & 0 \\ 0 & \chi_0^\| \end{pmatrix} + \frac{e^{-i(k-k')n_0}}{1 - v^2 \chi_0^\| \chi_0^\perp} \begin{pmatrix} v^2 \tilde{\chi}_k^\perp \chi_0^\| \tilde{\chi}_{k'}^\perp & v \tilde{\chi}_k^\perp \chi_0^\| \\ v \chi_0^\| \tilde{\chi}_{k'}^\perp & v^2 \chi_0^\| \chi_0^\perp \chi_0^\| \end{pmatrix}. \tag{23}$$

Since we are interested in optical excitation, the photon momentum conservation requires us to set $k = k' = 0$ in Supplementary Equation (23) and recast it to the following form

$$\overset{\leftrightarrow}{\alpha} = \begin{pmatrix} \tilde{\chi}_0^\perp & 0 \\ 0 & \chi_0^\| \end{pmatrix} + \frac{1}{1 - g^2 L_\perp^2 \chi_0^\perp \chi_0^\|} \begin{pmatrix} g^2 \tilde{\chi}_0^{\perp 2} \chi_0^\| & g \tilde{\chi}_0^\perp \chi_0^\| \\ g \tilde{\chi}_0^\perp \chi_0^\| & g^2 L_\perp^2 \chi_0^\perp {\chi_0^\|}^2 \end{pmatrix}, \tag{24}$$

where we denote $\overset{\leftrightarrow}{\alpha} = \overset{\leftrightarrow}{\tilde{\chi}}_{k=k'=0}$ and $\tilde{\chi}_0^{\perp} = L_{\perp}\tilde{\chi}_{k=0}^{\perp}$ by explicitly accounting for the oscillator strength scaling as $L_{\perp}$, and further introducing the normalized coupling $g = v/L_{\perp}$.

Next, we need to establish a relationship between $\tilde{\chi}_0^{\perp}(\omega)$ and $\chi_0^{\perp}(\omega)$, both entering Supplementary Equation (24). For this purpose, we specifically choose $\tilde{\chi}_{|n-m|}^{\parallel}(\omega)$ to be proportional to the local density of states in 1D systems,[7] *i.e.*,

$$\chi_{|n-m|}^{\perp}(\omega) = \frac{M_0}{L_{\perp}^2}\frac{e^{iz_{\perp}(\omega)|n|/\sqrt{\beta}}}{z_{\perp}(\omega)}, \tag{25}$$

quantity where $M_0 = |M_0|e^{i\varphi_0}$ is a complex number. For the density of states $\varphi_0 = \pi$, making $M_0 = i|M_0|$. For the Raman response, however, φ_0 is an adjustable parameter that can take values different from π. Within a tight binding approximation, β is the intertube exciton tunneling energy normalized per $L_{\perp}^2$ and $z_{\perp}(\omega) = \sqrt{\beta\,(\hbar\omega - E_0 - i\Gamma_0/2)}$. Direct evaluation gives

$$\begin{aligned} \tilde{\chi}_0^{\perp} &= L_{\perp}\tilde{\chi}_{k=0}^{\perp} = L_{\perp}\sum_{n-m=-L_{\perp}/2}^{L_{\perp}/2-1}\chi_{|n-m|}^{\perp} \\ &= \frac{M_0}{L_{\perp}}\sum_{n-m=0}^{L_{\perp}/2-1}\frac{e^{iz_{\perp}(\omega)|n-m|/\sqrt{\beta}}\left(1+e^{iz_{\perp}(\omega)/\sqrt{\beta}}\right)}{z_{\perp}} \\ &= \frac{M_0}{L_{\perp}}\frac{\left(1-e^{iz_{\perp}L_{\perp}/2\sqrt{\beta}}\right)\left(1+e^{iz_{\perp}/\sqrt{\beta}}\right)}{iz_{\perp}(1-e^{z_{\perp}/\beta})} \approx \frac{M_0}{z_{\perp}} = L_{\perp}^2\chi_0^{\perp}. \end{aligned} \tag{26}$$

Here the approximate equality is made by expanding the exponents up to the linear terms in $z_{\perp}$ which is small near the resonance. The last equality is obtained using Supplementary Equation (25) in which we set $n-m=0$. Thus,

$$\tilde{\chi}_0^{\perp}(\omega) = \frac{M_0}{\sqrt{\beta\,(\hbar\omega - E_0 - i\Gamma_0/2)}}. \tag{27}$$

Use of Supplementary Equation (27) allows us to recast Supplementary Equation (24) for the exciton polarizability tensor to the final compact form

$$\overset{\leftrightarrow}{\alpha} = \begin{pmatrix} \tilde{\chi}_0^{\perp} & 0 \\ 0 & \chi_0^{\parallel} \end{pmatrix} + \frac{1}{1-g^2\tilde{\chi}_0^{\perp}\chi_0^{\parallel}}\begin{pmatrix} g^2\tilde{\chi}_0^{\perp 2}\chi_0^{\parallel} & g\tilde{\chi}_0^{\perp}\chi_0^{\parallel} \\ g\tilde{\chi}_0^{\perp}\chi_0^{\parallel} & g^2\tilde{\chi}_0^{\perp}\chi_0^{\parallel 2} \end{pmatrix}. \tag{28}$$

Finally, the polarizability matrix can be represented as

$$\overset{\leftrightarrow}{\alpha}(\omega) = \begin{pmatrix} \alpha^{\perp}(\omega) & \alpha^{\times}(\omega) \\ \alpha^{\times}(\omega) & \alpha^{\parallel}(\omega) \end{pmatrix} \tag{29}$$

where the diagonal terms represent the intertube and intratube exciton polarizabilities, and the off-diagonal term represents the mixed exciton response

$$\alpha^{\perp}(\omega) = \frac{\tilde{\chi}_0^{\perp}(\omega)}{1 - g^2\tilde{\chi}_0^{\perp}(\omega)\chi_0^{\parallel}(\omega)}, \tag{30}$$

$$\alpha^{\parallel}(\omega) = \frac{\chi_0^{\parallel}(\omega)}{1 - g^2\tilde{\chi}_0^{\perp}(\omega)\chi_0^{\parallel}(\omega)}, \tag{31}$$

$$\alpha^{\times}(\omega) = \frac{g\tilde{\chi}_0^{\perp}(\omega)\chi_0^{\parallel}(\omega)}{1 - g^2\tilde{\chi}_0^{\perp}(\omega)\chi_0^{\parallel}(\omega)}, \tag{32}$$

respectively.

Supplementary Equations (30)–(32) provide components of the exciton (i.e. electronic) polarizability that parametrically depend on phonon coordinates. Connection with the Raman polarizability can be established via expansion of non-interacting inter- and intra-tube exciton polarizabilities into a power series in phonon coordinates and subsequent averaging over the phonon degrees of freedom. Such an expansion does not change the structure of Supplementary Equations (30)–(32). Therefore, the quantities entering Supplementary Equations (30)–(32) are identified as components of the Raman polarizability tensor in the analysis below and in the main text.

Supplementary Note 3: Resonance Raman excitation profile and generalized Fano lineshape.

The resonance Raman excitation profile is

$$\mathcal{I}(\omega) = \left\langle \left| \mathbf{e}_\mathrm{S} \cdot \overleftrightarrow{\alpha}(\omega) \cdot \mathbf{e}_\mathrm{L} \right|^2 \right\rangle, \tag{33}$$

where the angle brackets denote the rotationally averaged Raman tensor, $\overleftrightarrow{\alpha}$, multiplied by the polarization vectors for the excitation laser field $\mathbf{e}_\mathrm{L}$ and the scattered signal detection $\mathbf{e}_\mathrm{S}$. The Raman tensor introduced in Supplementary Equation (29) can be written in the form of a 3D matrix as

$$\overleftrightarrow{\alpha}(\omega) = \begin{pmatrix} \alpha^{\parallel}(\omega) & \alpha^{\times}(\omega) & 0 \\ \alpha^{\times}(\omega) & \alpha^{\perp}(\omega) & 0 \\ 0 & 0 & 0 \end{pmatrix}, \tag{34}$$

with the components given by Supplementary Equations (30)–(32). The tensor invariants such as isotropic invariant, symmetric anisotropy, and antisymmetric anisotropy read[8]

$$\bar{\alpha}^2(\omega) = \frac{1}{9}|\alpha^{\parallel}(\omega) + \alpha^{\perp}(\omega)|^2, \tag{35}$$

$$\bar{\gamma}_\mathrm{s}^2(\omega) = \frac{1}{2}\left(|\alpha^{\parallel}(\omega) - \alpha^{\perp}(\omega)|^2 + |\alpha^{\parallel}(\omega)|^2 + |\alpha^{\perp}(\omega)|^2\right) + 3|\alpha^{\times}(\omega)|^2, \tag{36}$$

$$\bar{\gamma}_\mathrm{as}^2 = 0, \tag{37}$$

respectively. The rotationally averaged Raman signal intensity for $\vec{e}_L \parallel \vec{e}_S$ can be expressed in terms of the tensor invariants as[8]

$$\mathcal{I}(\omega) = 45\bar{\alpha}^2(\omega) + 4\bar{\gamma}^2(\omega). \tag{38}$$

Direct substitution of Supplementary Equations (30)–(32) into Supplementary Equations (35) - (38) allows one to partition Supplementary Equation (38) as

$$\mathcal{I}(\omega) = \mathcal{I}_0(\omega)\mathcal{F}(\omega). \tag{39}$$

Here the background signal is

$$\mathcal{I}_0 = 5\left|\chi_0^{\parallel} + \tilde{\chi}_0^{\perp}\right|^2 + 2\left(|\chi_0^{\parallel} - \tilde{\chi}_0^{\perp}|^2 + |\chi_0^{\parallel}|^2 + |\tilde{\chi}_0^{\perp}|^2\right) + 12g^2|\tilde{\chi}_0^{\perp}(\omega)\chi_0^{\parallel}(\omega)|^2, \tag{40}$$

with the first two terms on the r.h.s. representing the direct contribution of the *non-interacting* intertube and intratube exciton states and the last term expressing the contribution of the coupled off-diagonal term in the Raman polarizability. The latter can be

neglected for relatively small coupling g values, making the background signal fully due to the non-interacting exciton contributions. Next we demonstrate that the lineshape function $\mathcal{F}(\omega)$ can be transformed to the form of a generalized Fano lineshape. With the help of Supplementary Equation (27) providing explicit representation of $\tilde{\chi}_0^{\perp}$, we find that

$$\begin{aligned}\mathcal{F}(\omega) &= \left|\frac{1}{1-g^2\tilde{\chi}_0^{\perp}(\omega)\chi_0^{\parallel}(\omega)}\right|^2 = \left|\frac{\sqrt{\beta\left(\hbar\omega-E_0-i\Gamma_0/2\right)}}{\sqrt{\beta\left(\hbar\omega-E_0-i\Gamma_0/2\right)}-g^2M_0\chi_0^{\parallel}(\omega)}\right|^2 \\ &= \left|\frac{\left[\sqrt{\beta\left(\hbar\omega-E_0-i\Gamma_0/2\right)}-g^2\mathrm{Re}\left\{M_0\chi_0^{\parallel}(\omega)\right\}\right]+\mathrm{Re}\left\{M_0\chi_0^{\parallel}(\omega)\right\}}{\sqrt{\beta\left(\hbar\omega-E_0-i\Gamma_0/2\right)}-g^2M_0\chi_0^{\parallel}(\omega)}\right|^2 .\end{aligned} \tag{41}$$

The last term in the r.h.s. of Supplementary Equation (41) can be written in the form of generalized Fano lineshape

$$\mathcal{F}(\omega) = \frac{(\epsilon+q)^2+\gamma_0^2}{\epsilon^2+1}, \tag{42}$$

with the following parameters

$$\epsilon = \mathrm{Re}\left\{\sqrt{\beta\left(\hbar\omega-E_0-i\Gamma_0/2\right)}-g^2M_0\tilde{\chi}_0^{\parallel}(\omega)\right\}/\gamma, \tag{43}$$

$$q = g^2\mathrm{Re}\left\{M_0\chi_0^{\parallel}(\omega)\right\}/\gamma, \tag{44}$$

$$\gamma = -\mathrm{Im}\left(\sqrt{\beta\left(\hbar\omega-E_0-i\Gamma_0/2\right)}-g^2M_0\tilde{\chi}_0^{\parallel}(\omega)\right), \tag{45}$$

$$\gamma_0 = \mathrm{Im}\sqrt{\beta\left(\hbar\omega-E_0-i\Gamma_0/2\right)}/\gamma, \tag{46}$$

representing reduced detuning from the Fano resonance, Fano lineshape asymmetry, total damping term including the decay of the intertube exciton states into the intratube continuum, and the ratio of the uncoupled intertube exciton damping rate to the total one, respectively.

Supplementary Note 4: Experimental data fit with Fano lineshape.

To fit the experimental data, we used the form of the total signal given by Supplementary Equation (39). Assuming negligible contribution of the intertube excitons to the background signal, *i.e.*, $\tilde{\chi}_0^{\perp}(\omega) \ll \chi_0^{\parallel}(\omega)$, the latter fully reduces to the response of the intratube excitons, *i.e.*,

$$\mathcal{I}_0(\omega) = \left|\chi_0^{\parallel}(\omega)\right|^2, \tag{47}$$

where the non-important pre-factor of 9 is dropped. We further use the Fano lineshape function in the form (see Supplementary Equations (41) and (42))

$$\mathcal{F}(\omega) \;=\; \left|\frac{1}{1-g^2\tilde{\chi}_0^{\perp}(\omega)\chi_0^{\parallel}(\omega)}\right|^2. \tag{48}$$

To evaluate the background signal, we employed the well established form of the Raman polarizability

$$\chi_0^{\parallel} \;=\; \frac{M_1}{\hbar\omega - E_{22}^{\mathrm{S}} - i\Gamma_{22}/2} + \frac{M_2}{\hbar\omega - E_{22}^{\mathrm{S}} - E_{\mathrm{ph}} - i\Gamma_{22}/2}, \tag{49}$$

parametrized for the $(6,5)$-SWCNT. Related parameters such as intratube exciton energy, E_{22}^{S}, associated broadening, Γ_{22}, and the non-Condon factor, $C = (M_1 + M_2)/(M_1 - M_2)$, are listed in Table 1 of the main text. The phonon energy, E_{ph}, was set to its experimentally measured values listed in Supplementary Table 2 (in cm^{-1} units) for the RBM and G^+ phonons as ω_{RBM} and ω_{G^+}, respectively. Furthermore, we adopted the intertube exciton polarizability in the form of Supplementary Equation (27) explicitly accounting for its dependence on the absolute value of the matrix element $|M_0|$ and its phase φ_0, *i.e.*,

$$\tilde{\chi}_0^{\perp}(\omega) = \frac{|M_0|\, e^{i\varphi_0}}{\sqrt{\beta\,(\hbar\omega - E_0 - i\Gamma_0/2)}}. \tag{50}$$

Substitution of Supplementary Equation (49) and (50) into Supplementary Equation (48) reveals *four* fitting parameters such as E_0, Γ_0, the ratio $M = g^2|M_0|/\sqrt{\beta}$, and the phase φ_0. Supplementary Figure 3 shows the experimental data fit with this model and Supplementary Table 3 lists the parameters obtained from the data fit.

Supplementary Note 5: Fano interference.

Under the assumption of dark intertube exciton states, the intratube exciton polarizability can be written using the scattering matrix formalism [*i.e.*, Supplementary Equations (22) and (28)] as

$$\alpha^{\|}(\omega) \;=\; \chi_0^{\|}(\omega) + \chi_0^{\|}(\omega)T^{\|}(\omega)\chi_0^{\|}(\omega), \tag{51}$$

with the scattering operator matrix element

$$T^{\|}(\omega) \;=\; \frac{g^2\chi_0^{\perp}(\omega)}{1 - g^2\tilde{\chi}_0^{\perp}(\omega)\chi_0^{\|}(\omega)}. \tag{52}$$

This matrix element describes the intratube exciton scattering by the intertube exciton. Note that substitution of Supplementary Equation (52) into Supplementary Equation (51) and straightforward re-arrangement of terms shows that Supplementary Equation (52) and Supplementary Equation (51) reproduce Supplementary Equation (31) exactly.

Taking the absolute value of $\alpha^{\|}(\omega)$ (Supplementary Equation (51)) and further partitioning the background signal $\mathcal{I}_0 = |\chi_0^{\|}(\omega)|^2$, one can write down the Fano lineshape function as

$$\mathcal{F}(\omega) \;=\; \left|1 + \left|T^{\|}(\omega)\chi_0^{\|}(\omega)\right| e^{i\phi(\omega)}\right|^2 = 1 + 2\left|T^{\|}(\omega)\chi_0^{\|}(\omega)\right| \cos\phi(\omega) + \left|T^{\|}(\omega)\chi_0^{\|}(\omega)\right|^2. \tag{53}$$

Supplementary Equation (53) clearly shows that the Fano lineshape forms as a result of the interference between the unperturbed response of the intratube excitons (unity term) and the dark exciton scattering term $T^{\|}(\omega)\chi_0^{\|}(\omega)$ whose absolute value and phase, $\phi(\omega)$ are introduced explicitly.

Supplementary Note 6: Fano lineshape of two coupled classical oscillators.

Let us assume that the response of the intratube (bright) and the intertube (dark) excitons are approximated by the damped classical oscillators

$$\chi_0^{\parallel}(\omega) = \frac{1}{{\omega_\mathrm{b}}^2 + i\gamma_\mathrm{b}\omega - \omega^2}, \tag{54}$$

$$\tilde{\chi}_0^{\perp}(\omega) = \frac{1}{{\omega_\mathrm{d}}^2 + i\gamma_\mathrm{d}\omega - \omega^2}, \tag{55}$$

where we denoted ω_b (ω_d) and γ_b (γ_d) the bright (dark) oscillator central frequency and damping rate, respectively. Furthermore, we assume that $\gamma_\mathrm{d} \ll \gamma_\mathrm{b}$. Substitution of Supplementary Equations (54) and (55) into Supplementary Equation (53) along with Supplementary Equation (52), results in the Fano lineshape function given by Supplementary Equation (42) with the parameters

$$\tag{56}$$

$$q = \frac{\omega_\mathrm{b}^2 - \omega_\mathrm{d}^2}{\gamma_\mathrm{b}\omega_\mathrm{d}}, \tag{57}$$

$$\gamma = \frac{g^2\gamma_\mathrm{b}\omega_\mathrm{d}}{(\omega_\mathrm{b}^2 - \omega_\mathrm{d}^2)^2 + \gamma_\mathrm{b}^2\omega_\mathrm{d}^2} \tag{58}$$

$$\gamma_0 = \gamma_\mathrm{d}\omega_\mathrm{d}/\gamma. \tag{59}$$

Supplementary Equation (43) along with Supplementary Equations (56)– (59) exactly reproduces the two oscillator model developed in Ref. [9].

Supplementary References

---

[1] O'connell, M. J. *et al.* Band gap fluorescence from individual single-walled carbon nanotubes. *Science* **297**, 593–596 (2002).

[2] Wang, F. *et al.* Interactions between individual carbon nanotubes studied by Rayleigh scattering spectroscopy. *Phys. Rev. Lett.* **96**, 167401 (2006).

[3] Crochet, J. J., Sau, J. D., Duque, J. G., Doorn, S. K. & Cohen, M. L. Electrodynamic and excitonic intertube interactions in semiconducting carbon nanotube aggregates. *ACS Nano* **5**, 2611–2618 (2011).

[4] Naumov, A. V., Ghosh, S., Tsyboulski, D. A., Bachilo, S. M. & Weisman, R. B. Analyzing absorption backgrounds in single-walled carbon nanotube spectra. *ACS Nano* **5**, 1639–1648 (2011).

[5] Torrens, O. N., Zheng, M. & Kikkawa, J. M. Energy of k-momentum dark excitons in carbon nanotubes by optical spectroscopy. *Phys. Rev. Lett.* **101**, 157401 (2008).

[6] Duque, J. G. *et al.* Violation of the Condon approximation in semiconducting carbon nanotubes. *ACS Nano* **5**, 5233-5241 (2011).

[7] Economou, E. N. *Green's Functins in Quantum Physics* (Springer, New York, 2006).

[8] Reich, S., Thomsen, C. & Maultzsch, J. *Carbon Nanotubes. Basic Concepts and Physical Properties* (WILEY-VCH, Darmstadt, 2005).

[9] Gallinet, B. & Martin, O. J. Ab initio theory of Fano resonances in plasmonic nanostructures and metamaterials. *Phys. Rev. B* **83**, 235427 (2011).
